# LSY-2 is essential for maintaining the germ-soma distinction in *C. elegans*

**DOI:** 10.1007/s13238-015-0173-1

**Published:** 2015-06-07

**Authors:** Long Lin, Yuping Li, Libo Yan, Gangming Zhang, Yu Zhao, Hong Zhang

**Affiliations:** College of Life Sciences, Beijing Normal University, Beijing, 100875 China; National Institute of Biological Sciences, Beijing, 102206 China; State Key Laboratory of Biomacromolecules, Institute of Biophysics, Chinese Academy of Sciences, Beijing, 100101 China

**Keywords:** P granules, soma, *lsy-2*, *C. elegans*

## Abstract

The mechanisms that specify and maintain the characteristics of germ cells during animal development are poorly understood. In this study, we demonstrated that loss of function of the zinc-finger gene *lsy-2* results in various somatic cells adopting germ cells characteristics, including expression of germline-specific P granules, enhanced RNAi activity and transgene silencing. The soma to germ transformation in *lsy-2* mutants requires the activities of multiple chromatin remodeling complexes, including the MES-4 complex and the ISW-1 complex. The distinct germline-specific features in somatic cells and the gene expression profile indicate that LSY-2 acts in the Mec complex in this process. Our study demonstrated that *lsy-2* functions in the maintenance of the soma-germ distinction.

## INTRODUCTION

Germ cells are specified at an early embryonic stage in most sexually reproducing animals. In *C. elegans*, germline lineage is generated through each of four unequal divisions in which a blastomere generates one somatic founder cell and one germline blastomere (Strome, 2005). The primordial germ cell P4 then divides equally at the embryonic stage, giving rise to two germ precursor cells Z2 and Z3, which remain quiescent during embryogenesis and proliferate throughout larval development to produce germ cells (Strome, 2005). Germ P granules, which are aggregates of RNA and proteins, are maternally contributed and segregated exclusively into the germline lineages (Strome, 2005). P granules are synthesized in all of the descendants of P4 with the exception of mature sperm and are associated with the outer surface of the nuclear envelope (Hird et al., 1996). P granules contain constitutive components, which associate with P granules during all developmental stages, and also transient components, which interact with P granules in germline blastomeres and disappear in Z2 and Z3 (Strome, 2005). Constitutive components include the RGG box containing RNA binding proteins PGL-1 and PGL-3 and the RNA helicases GLH-1 to GLH-4 (Gruidl et al., 1996; Kawasaki et al., 1998; Kuznicki et al., 2000). During *C. elegans* embryogenesis, PGL-1 and PGL-3 are degraded by autophagy in somatic cells (Zhang et al., 2009). In autophagy mutants, PGL-1 and PGL-3 colocalize and accumulate into aggregates in somatic cells (Zhang et al., 2009).

Establishment of somatic and germ precursor cells during early embryogenesis in *C. elegans* requires maternally distributed proteins, including PIE-1, MEX-1, MEX-3, and POS-1, which are also associated with P granules during the first several cell divisions and disappear in the daughters of the germ precursor P4 cells (Mello et al., 1996; Guedes and Priess, 1997; Tabara et al., 1999). PIE-1, MEX-1, and POS-1 prevent the germline blastomere from adopting somatic fates, while MEX-3 prevents certain somatic blastomeres from adopting a germ blastomere fate (Draper et al., 1996). Maintenance of germ cell fate during animal development involves multiple antagonistic chromatin remodeling complexes (Cui et al., 2006). Mutations in the SynMuv B genes, including the components of the *C. eleganslin-35* Rb pathway and the MEP-1/LET-418/HAD-1 NURD complex, lead to the ectopic expression of germline traits by somatic cells (Unhavaithaya et al., 2002; Wang et al., 2005; Kunert et al., 2009). SynMuv B genes function redundantly with SynMuv A genes in preventing extra cells from adopting the vulval fate (Fay and Yochem, 2007). Animals defective in both SynMuv A and SynMuv B genes display a multi-vulva (Muv) phenotype. According to distinct germline-specific features in somatic cells and the profile of misexpressed small RNA and P granule genes, SynMuv B genes have been classified into three classes, representing three functional complexes: a LIN-35/RB-containing core complex (DRM), a heterochromatin complex and a SUMO-recruited Mec complex (Wu et al., 2012; Tables 1, 2). The soma to germ transformation in these mutants is antagonized by the ISW-1 complex and the MES-4 complex (Cui et al., 2006; Stielow et al., 2008), which appear to modulate the active chromatin structures. How these complexes are targeted to germline-specific genes and faithfully maintain the repressive state of these genes in somatic cells is unknown.

**Table 1:** Distinct classes of SynMuv B genes

Class/Gene name	Measured phenotypes	
	Transgene silencing	Somatic PGL-1 granule morphology
DRM class (*lin-52*, *dpl-1*, *efl-1*, *lin-35*, *lin-9*, *lin-54*, *lin-37*, *lin-53*, *lin-15b*)	Yes	Large and sparse
SUMOylation and Mec complex (*mep-1*, *let-418*, *smo-1*, *uba-2*, *ubc-9*)	Yes	Small and dense
Heterochromatin class (*hpl-2*, *lin-13*, *lin-61*, *met-2*, *met-1*, *lin-65*)	No	Small and dense
*lsy-2*	Yes	Small and dense

**Table 2:** Differential misexpression of RNAi factors and P granule genes among different SynMuv B classes mutants and *lsy-2* mutants

Target category	Gene name	DRM inactivations	Heterochromatin inactivations	Mec&SUMO inactivations	*lsy-2* inactivation
Germline-enriched common targets	*pgl-1*	Upregulated	Upregulated	Upregulated	Upregulated
	*glh-1*				
	*pgl-3*				
	*wago-9*				
Ubiquitous common targets	*C04F12.1*	Upregulated	Upregulated	Upregulated	Upregulated
	*sago-2*				
	*rrf-2*				
Germline-enriched DRM targets	*spn-4*	Upregulated	Little change	Little change	Little change
	*mut-2*				
	*drh-3*				
	*rde-4*				
Germline-enriched SynMuv B heterochromatin & Mec targets	*wago-1*	Little change	Upregulated	Upregulated	Upregulated
	*wago-2*				

*lsy-2*, encoding a C2H2 zinc-finger transcription factor, is involved in specifying the left-right asymmetry of the ASE neurons (Johnston and Hobert, 2005). In this study, we found that loss of function of *lsy-2* causes ectopic expression of germ cell characteristics in various somatic cells, including the perinuclear localization of P granules, enhanced RNAi and transgene silencing. The phenotype resembles that in mutants for SUMO-recruited Mec complex factors MEP-1 and LET-418 (Stielow et al., 2008; Wu et al., 2012). The Mec complex also functions in specification of the ASE fate. Our study demonstrates that *lsy-2* genetically interacts with the MEP-1/LET-418 complex and is involved in maintaining soma-germ distinction and ASE cell fate specification.

## RESULTS

### Loss of function of *lsy-2* leads to transformation of somatic cell to germ cell fate

To study how somatic- and germ cell-specific fates are specified, we screened a library of bacterial clones expressing dsRNAs designed to individually inactivate 16,749 genes (targeting about 87% of the predicted genes). We looked for gene inactivations that cause ectopic expression of the P granule-specific reporter, *gfp*::*pgl-1*, in somatic cells of larval animals. In addition to components of the *lin-35* Rb pathway, including *lin-35*, *lin-53*, *hpl-2*, *lin-9*, *dpl-1* and *lin-52*, and the Mec complex component *mep-1*, we identified that inactivation of the zinc-finger transcription factor *lsy-2* and components of the sumoylation pathway, including *smo-1*, *ubc-9*, and *uba-2*, caused ectopic accumulation of GFP::PGL-1 granules in somatic cells.

**Figure 1:**
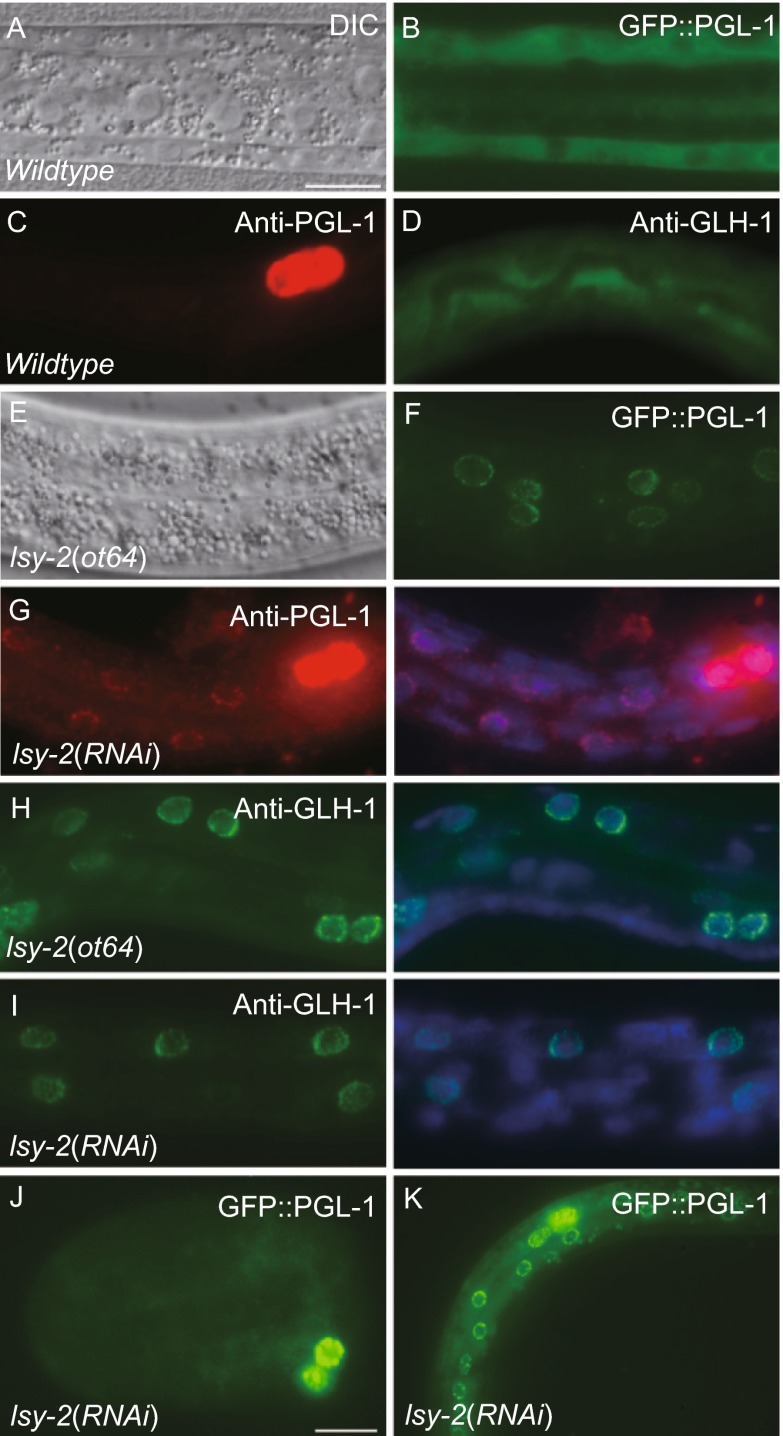
**Loss of function of** ***lsy-2*** **results in ectopic expression of GFP::PGL-1 in somatic cells**. (A and B) In wild type larvae, GFP::PGL-1 is weakly expressed and diffusely localized in the cytoplasm. *gfp::pgl-1* is driven by the germline-specific *pie-1* promoter and is non-specifically expressed in hypodermal cells from late embryonic stages, but GFP::PGL-1 fails to assemble into P granules, probably due to lack of other P-granule components. (A) DIC image of the animal shown in (B). (C and D) Endogenous PGL-1 and GLH-1, detected by the corresponding antibodies, do not form granules in somatic cells in wild type animals. The two cells with strong staining signal in (C) are the germline cells. (E and F) In *lsy-2*(*ot64*) mutants, GFP::PGL-1 forms distinct granules surrounding the nuclei in hypodermal cells. (E) DIC image of the animal shown in (F). (G–I) Immunostaining assays reveal that endogenous PGL-1 (G) and GLH-1 (H and I) are ectopically expressed and form granules in somatic cells in *lsy-2* mutants. (J and K) In *lsy-2* mutants, ectopic GFP::PGL-1 granules are not detected at embryonic stages (J), but become evident in L1 larvae (K). The two cells with strong GFP signals in (J) are the germline precursor cells Z2 and Z3

Loss of function of *lsy-2* caused ectopic expression of GFP::PGL-1 in various somatic cells, including hypodermal cells and intestinal cells. As in germ cells, GFP::PGL-1 formed distinct granules surrounding the nuclei in somatic cells in *lsy-2* mutants (Fig. 1A–C and 1G). *lsy-2* encodes a zinc-finger protein (Johnston and Hobert, 2005). The genetic null mutant *lsy-2*(*ot64*) also showed somatic misexpression of GFP::PGL-1 (Fig. 1E and 1F). To determine whether endogenous P-granule components were also ectopically expressed, we stained *lsy-2* mutant animals with anti-PGL-1 and anti-GLH-1 antibodies and found that endogenous PGL-1 and GLH-1 were also ectopically expressed and formed perinuclear granules in somatic cells (Fig. 1C, 1D, and 1G–I).

We further examined the temporal expression pattern of GFP::PGL-1 in *lsy-2* somatic cells. We found that ectopic GFP::PGL-1 or endogenous PGL-1 granules in *lsy-2* mutants were not formed until the early L1 larval stage, when the germ precursor cells Z2 and Z3 start to proliferate (Fig. 1J and 1K). Therefore, *lsy-2* is not involved in *de novo* establishment of the soma-germ distinction in *C. elegans*, but appears to be required for its maintenance.

### *lsy-2* mutants show enhanced RNAi interference and display transgene silencing

**Figure 2:**
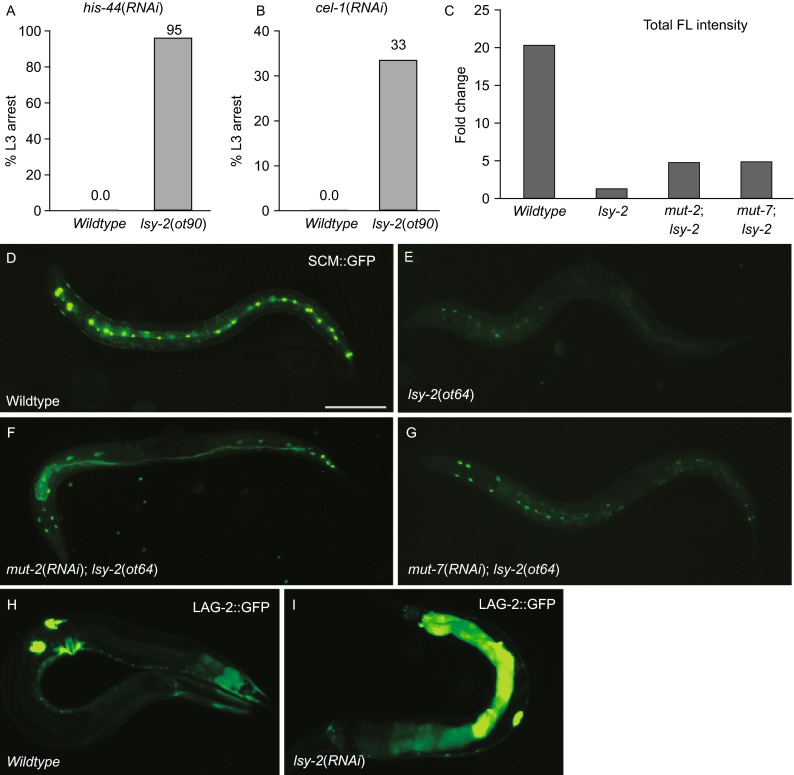
***lsy-2*** **mutants show enhanced RNAi interference and display transgene silencing**. (A and B) Percentage of wild-type and *lsy-2*(*ot90*) mutant animals that were arrested at the L3 stage after feeding with RNAi bacterial clones of *his-44* (A) and *cel-1* (B). (C) Quantification of total fluorescence intensity of SCM::GFP in different genetic backgrounds. (D and E) Compared with wild-type animals (D), the expression level of SCM::GFP is dramatically decreased in *lsy-2*(*ot64*) mutants (E). (F and G) Simultaneous inactivation of *mut-2* (F) and *mut-7* (G) restores the expression of SCM::GFP in *lsy-2*(*ot64*) mutants. (H and I) Compared with wild type worms (H), loss of function of *lsy-2* causes ectopic expression of LAG-2::GFP (I)

We next determined whether *lsy-2* mutants exhibit other characteristics associated with the germline. Germ cells exhibit elevated RNAi efficiency (Sijen and Plasterk, 2003; Robert et al., 2005). We determined whether mutations in *lsy-2* enhanced the RNAi efficiency in somatic cells. Feeding of bacterial clones of *his-44* and *cel-1* had little effect on wild-type animals, but led to 95% (*n* = 106) and 33% (*n* = 124) larval arrest in *lsy-2*(*ot90*) mutants, respectively, indicating that the RNAi efficiency is enhanced in *lsy-2* mutants (Fig. 2A and 2B).

Germ cells are more protected than somatic cells from foreign genetic elements, such as multicopy transgenes (Sijen and Plasterk, 2003; Robert et al., 2005). Therefore, transgenes introduced by microinjection into the germline are quickly silenced, a process mechanistically related to RNAi (Sijen and Plasterk, 2003; Robert et al., 2005). SCM::GFP is strongly expressed in seam cells. We found that expression of SCM::GFP was significantly reduced in *lsy-2* mutants (Fig. 2C–E). The number of seam cells expressing SCM::GFP was decreased in *lsy-2* mutants (Fig. 2D and 2E). The SCM::GFP fluorescence intensity (SCM::GFP expressed in all seam cells of one worm) was also measured and we found that the intensity in wild-type worms was about twenty times higher than that in *lsy-2* mutants (Fig. 2C). Simultaneous inactivation of *mut-2* or *mut-7*, essential components in the RNAi pathway, partially restored the expression of SCM::GFP in *lsy-2* mutants (Fig. 2C, 2F, and 2G). Therefore, somatic cells in *lsy-2* mutants display characteristics of germ cells.

### *lsy-2* functions similar to Mec class genes in repressing expression of germline genes in somatic cells

Based on phenotypic differences and the different profile of misexpressed RNAi factors and P-granule components in somatic cells, SynMuv B genes can be divided into three distinct classes: a DRM core complex, a SUMO-recruited Mec complex, and a SynMuv B heterochromatin complex (Wu et al., 2012; Table 1). The target genes upregulated in different SynMuv B mutants have been categorized into germline-enriched common targets, ubiquitous common targets, germline-enriched DRM targets, and germline-enriched SynMuv B heterochromatin and Mec targets. We showed above that *lsy-2* mutants exhibited transgene silencing (Fig. 2C–G). The PGL-1 granules that were somatically misexpressed in intestine and hypodermal cells were small and densely distributed around the nuclei (Fig. 1E, 1F, and 1K). Mutations in *lsy-2* also caused ectopic expression of LAG-2::GFP in the intestine, a phenotype associated with loss of function of Mec class genes (Fig. 2H and 2I) (Poulin et al., 2005; Cui et al., 2006). Thus, *lsy-2* mutants show similar phenotypes to those in Mec complex mutants.

**Figure 3:**
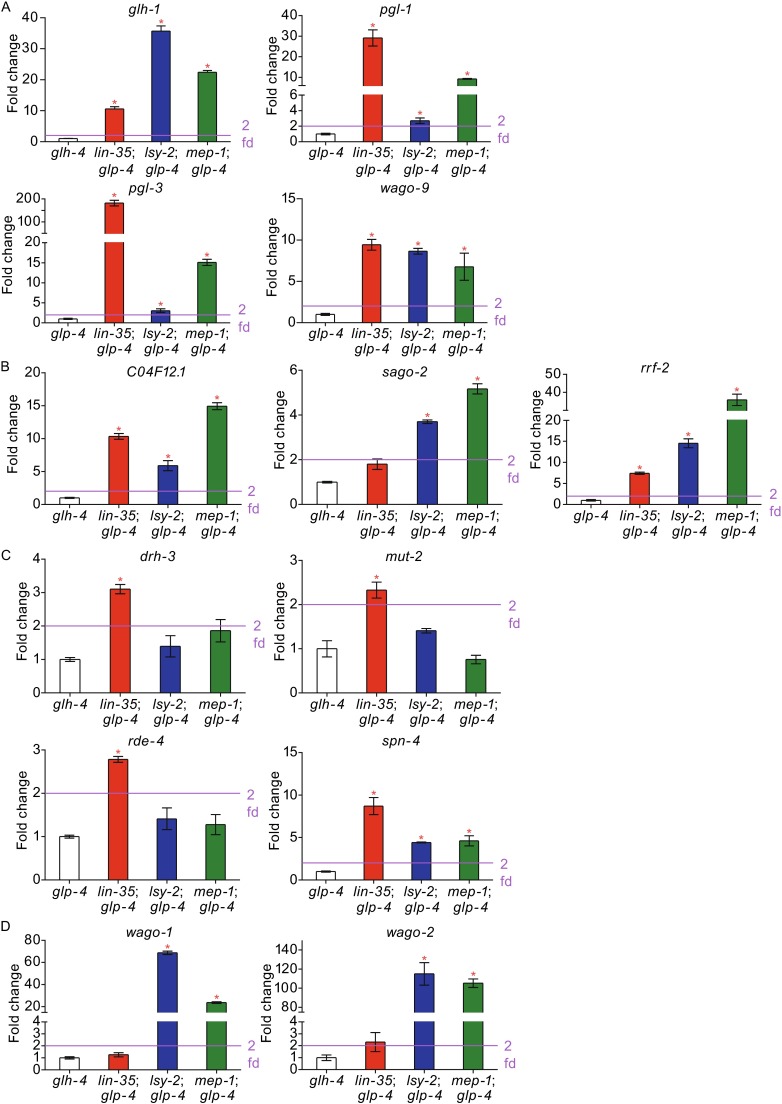
**Expression pattern of SynMuv B target genes in** ***lsy-2*** **mutants**. (A–D) Target genes are categorized into germline-specific common targets (A), ubiquitously expressed common targets (B), DRM-specific targets (C), and SynMuv B heterochromatin and Mec-specific targets (D). L4 stage *glp-4* animals with the indicated RNAi treatments were used in real-time PCR assays. Expression levels are normalized to that of *glp-4* worms, which is set to 1. Asterisks indicate that the expression change is greater than 2-fold and the *P*-value is less than 0.05 in two-tailed tests

We next tested *lsy-2* mutants for the expression levels of the 13 genes that are differentially expressed in distinct SynMuv B classes (Wu et al., 2012). To avoid any possible contamination of somatic cells by the germline, we used *glp-4*(*bn2*) temperature sensitive mutants, which have no germline when cultured at 25°C. According to the real-time PCR results, germline-enriched common targets, ubiquitous common targets and, more significantly, germline-enriched SynMuv B heterochromatin and Mec-specific targets were dramatically upregulated upon inactivation of *lsy-2*, while germline-enriched DRM-specific targets showed little change (Fig. 3A–D; Table 2), indicating that *lsy-2* is related to either SynMuv B heterochromatin or Mec class genes. Taken together, *lsy-2* acts similarly to Mec class genes in maintaining the germ-soma distinction.

### Inactivation of *lsy-2* in SynMuv mutants causes no SynMuv phenotype

The role of *lsy-2* in repressing the expression of germline cell fates in somatic cells resembles that of SynMuv B genes. SynMuv B genes function redundantly with SynMuv A genes in repressing the vulva cell fate. Animals defective in both SynMuv A and SynMuv B genes display a multivulva (Muv) phenotype. We thus examined whether loss of function of *lsy-2* causes Muv phenotype. RNAi inactivation of *lsy-2* in SynMuv mutants, including the SynMuv B mutants *lin-35*, *lin-9*, *lin-15B*, and *hpl-2* and the SynMuv A mutants *lin-8*, *lin-15A*, *lin-56*, and *lin-38*, caused no SynMuv phenotype. However, 37% of *lsy-2* mutants (*n* = 40) had multivulva phenotype when raised at 20°C. Similarly, *smo-1* mutants also show a weak Muv phenotype (Broday et al., 2004; Leight et al., 2005). Thus, Muv phenotype of *lsy-2* is not enhanced by inactivation of other SynMuvs.

### *lsy-2* synergistically interacts with the LIN-35/Rb-containing DRM complex in specifying somatic cell fate

We next determined the relationship between *lsy-2* and other SynMuv B genes in repressing the expression of germline cell fates. Neither *lsy-2* nor *lin-35* single mutations caused a larval arrest phenotype, while 100% of *lin-35*; *lsy-2* double mutants (*n* = 93) arrested at the L1 stage. The synthetic lethal phenotype was also observed between *lsy-2* and mutations in other DRM class genes, including *lin-15B*, *dpl-1*, *lin-53*, *lin-37*, and *lin-9* (data not shown). This suggests that the DRM complex and *lsy-2* may act in concert to regulate the expression of one or more critical targets during development.

**Figure 4:**
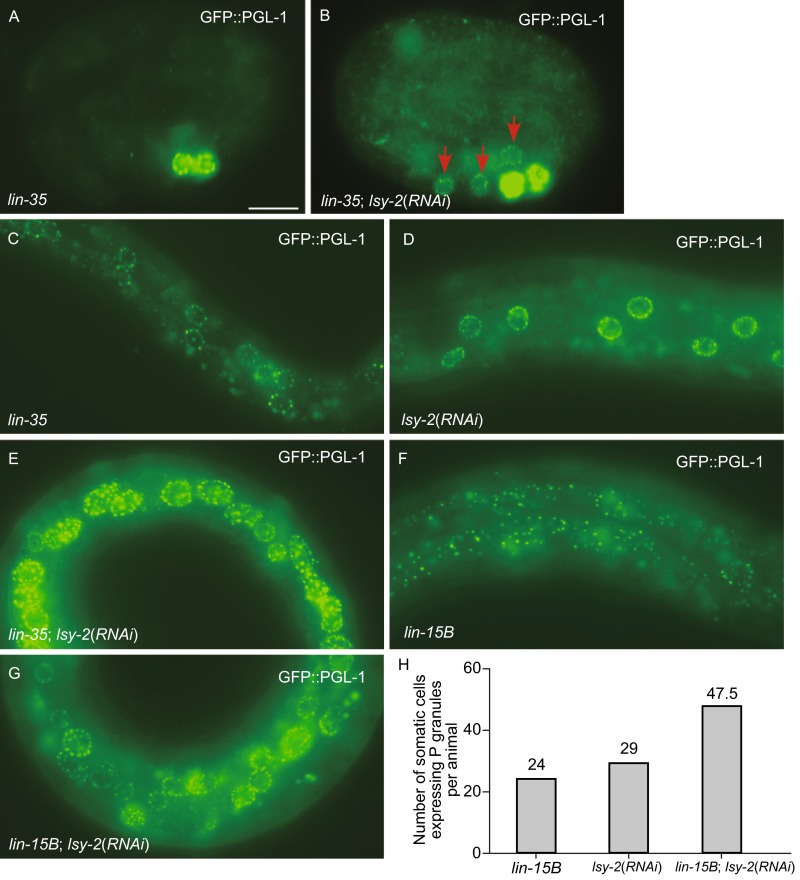
**Synergistic interaction between** ***lsy-2*** **and** ***lin-35*** **or** ***lin-15B*** **in repressing the expression of P granules in somatic cells**. (A and B) In *lin-35* mutants, ectopic GFP::PGL-1 granules are not detected at embryonic stages (A), but become evident in multiple extra cells (highlighted with red arrows) at the 4-fold stage in *lin-35*; *lsy-2*(*RNAi*) double mutants (B). (C–E) Compared with *lin-35* (C) and *lsy-2* (D) single mutants, the expression level of GFP::PGL-1 is dramatically increased in *lin-35*; *lsy-2*(*RNAi*) double mutants (E). (F and G) Compared with *lin-15B* (F), expression level of GFP::PGL-1 is increased in *lin-15B*; *lsy-2*(*RNAi*) double mutants (G). (H) Number of somatic cells per animal expressing GFP::PGL-1 in different mutants

We next examined P granule formation in *lin-35*; *lsy-2* double mutants. In *lsy-2* and *lin-35* single mutants, P granules were not ectopically expressed until the L1 larval stage (Figs. 1J, 1K, and 4A). In contrast, the onset of ectopic expression of P granules in *lin-35*; *lsy-2* double mutants occurred much earlier. 20% (*n* = 15) and 74% (*n* = 19) of double mutant embryos contained multiple extra cells expressing perinuclear P granules at the 2-fold and 3-fold stage, respectively (Fig. 4B). Moreover, the number of somatic cells expressing P granules was dramatically increased in *lin-35*; *lsy-2* mutant larvae (Fig. 4C–E). We also examined P granule formation in *lin-15B* mutants after *lsy-2* RNAi injection. The number of somatic cells expressing P granules was dramatically increased in *lin-15B* larval mutants after *lsy-2* RNAi injection (Fig. 4F–H). Thus, *lsy-2* and the LIN-35/Rb*-*containing DRM complex function in parallel to repress the expression of germ cell traits in somatic cells.

### Formation of P granules in *lsy-2* somatic cells requires the activities of multiple chromatin remodeling complexes

**Figure 5:**
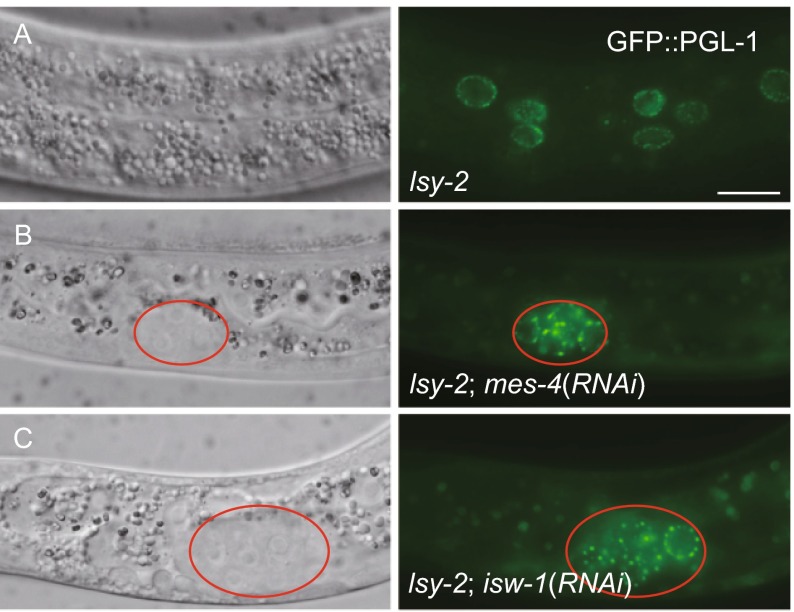
**Formation of P granules in** ***lsy-2*** **somatic cells requires the activities of multiple chromatin remodeling complexes**. (A) Misexpressed GFP::PGL-1 forms perinuclear granules in hypodermal cells in *lsy-2*(*ot64*) mutants. (B and C) Loss of function of *mes-4* (B) and *isw-1* (C) suppresses ectopic expression of GFP::PGL-1 in *lsy-2*(*ot64*) mutants. Germline cells are highlighted with red circles

Formation of perinuclear P granules in somatic cells in the SynMuv B mutants requires the activity of chromatin remodeling complexes, including the MES-4 complex and the ISWI complex (Unhavaithaya et al., 2002; Cui et al., 2006). We investigated the role of several chromatin remodeling complexes in the ectopic expression of P granules in *lsy-2* mutants. We found that inactivation of the NuA4 complex, the ISW1 complex, and the COMPASS complex dramatically decreased the formation of P granules in somatic cells in *lsy-2*(*ot64*), while mutations in components of the SWR1 complex and the SWI/SNF complex had no obvious effect (Fig. 5A–C and Table 3). Thus, various chromatin-remodeling complexes can regulate the expression of different P-granule components or function non-redundantly in regulation of common P-granule components.

**Table 3:** Summary of the suppression of the formation of ectopic P granules in *lsy-2* somatic cells by inactivation of various chromatin-remodeling complexes

Complex	Gene	Suppression of *lsy-2* (%)*	*n*
NuA4 complex	*C34B7.4*	N	>40
	*gfl-1*	Y (100%)	23
	*mrg-1*	Y (97%)	30
	*ekl-4*/*Y105E8A.17*	N	>40
	*ZK1127.3*	Y (75%)	8
SWR1 complex	*C08B11.6*	N	>40
	*C17E4.6*	N	>40
	*CD4.7*	N	>40
COMPASS	*C14B1.4*	Y (90%)	21
	*dpy-30*	Y (100%)	27
	*hcf-1*	N	>40
	*mes-4*	Y (89%)	82
ISW1 complex	*isw-1*	Y (87%)	28
	*nurf-1*	Y	22
SWI/SNF complex	*lin-49*	N	>40
	*lin-59*	N	>40

*The percentage of *lsy-2* animals in which the formation of ectopic P granules was suppressed by inactivation of each gene is indicated in parenthesis. N: no suppression was observed; Y: suppression

### Mutations in *lsy-2* and Mec class genes result in a defect in ASE asymmetry specification

**Figure 6:**
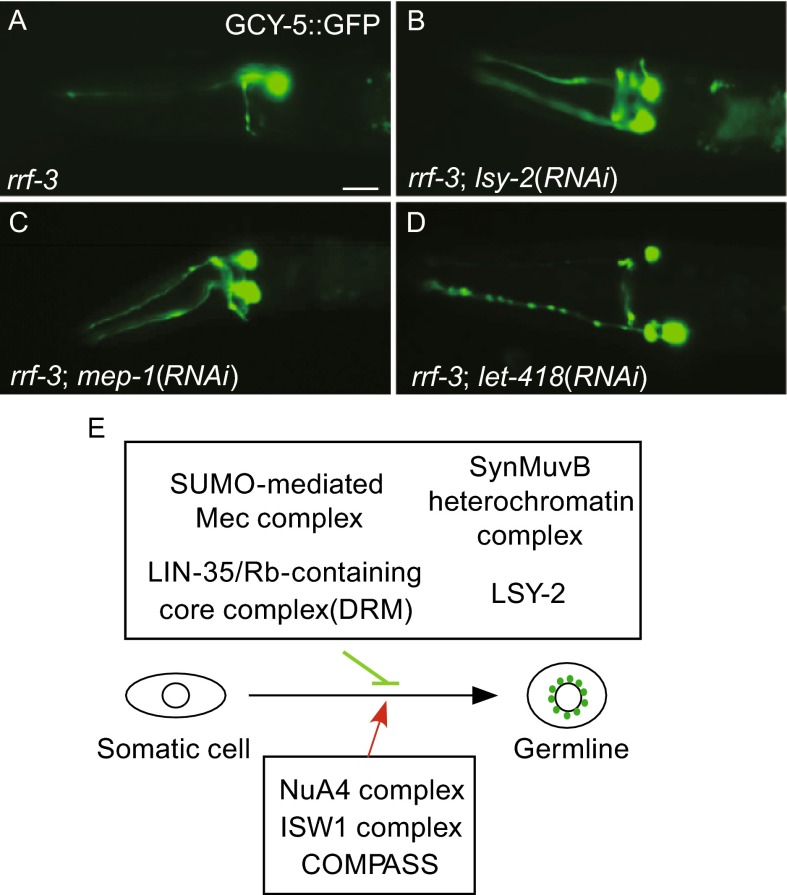
**Mutations in** ***lsy-2*** **and Mec class genes result in a defect in ASE asymmetry specification**. (A) In *rrf-3* animals, GCY-5::GFP is specifically expressed in ASER neurons. (B–D) RNAi inactivation of *lsy-2* (B), *mep-1* (C), and *let-418* (D) caused ectopic expression of GCY-5::GFP in ASEL neurons. (E) Model for antagonistic roles of SynMuvB genes and chromatin remodeling genes in the regulation of germline-specific gene expression

LSY-2 is involved in specifying the asymmetry of the ASE neurons (Johnston and Hobert, 2005). Two taste receptor neurons, ASE left (ASEL) and ASE right (ASER) are morphologically bilaterally symmetric, but they display a left/right asymmetric function and express a distinct set of chemosensory receptors (Sarin et al., 2007; Ortiz et al., 2009). In *lsy-2* mutants, the ASEL neuron expresses the ASER-specific terminal fate markers and adopts the ASER cell fate (Fig. 6A and 6B). We showed above that LSY-2 genetically acts in the Mec complex to maintain the soma-germ distinction. We determined whether inactivation of Mec complex components also results in ASE asymmetry defects. As neurons are refractory to RNAi, we used RNAi-sensitive *rrf-3* animals. In *rrf-3* animals, the ASER marker GCY-5::GFP is exclusively expressed in ASER (Fig. 6A). After RNAi inactivation of *mep-1* and *let-418*, 25% (*n* = 32) and 12% (*n* = 40) animals showed ectopic expression of GCY-5::GFP in ASEL, respectively (Fig. 6C and 6D). Inactivation of the DRM class genes *lin-35* and *lin-15B* and the heterochromatin class genes *hpl-2* and *lin-65* failed to cause ectopic expression of GCY-5::GFP (data not shown). Misexpression of GCY-5::GFP in ASEL was also reported in *smo-1* mutants (Poole et al., 2011). Therefore, Mec complex mutants also exhibit the ASE asymmetry specification defect, further supporting the idea that *lsy-2* is genetically related to Mec class genes.

## DISCUSSION

In this study, we demonstrated that loss of function of *lsy-2* causes somatic cells to display some germline-specific features, including ectopic expression of P-granule components, enhanced RNAi efficiency, and transgene silencing. Consistent with these observations, mutations in *lsy-2* results in the ectopic expression of a set of germ cell-specific genes in somatic cells (Fig. 6E). *lsy-2* has previously been shown to be involved in the specification of the left-right asymmetry of ASE neurons by regulating the cell-type specific expression of *lsy-6* miRNA (Johnston and Hobert, 2005). Loss of function of *lsy-6* and other components involved in specification of ASEL fate, including *lin-49*, *lim-6*, and *ceh-36*, does not lead to the ectopic expression of P granules in somatic cells, indicating that *lsy-2* functions in separate pathways in determining left-right asymmetry and soma-germ distinction.

How does *lsy-2* function in these processes? *lsy-2* encodes a zinc-finger protein that is distantly related to SP1/KLF family transcription factors (Kaczynski et al., 2003; Johnston and Hobert, 2005). The repressed state of germ cell-specific genes is maintained over cell divisions in somatic cells during *C. elegans* development. During late embryogenesis and larval stages, this repressive state requires multiple functional antagonistic chromatin remodeling complexes. The LIN-35/Rb complex and the Mec complex are involved in maintaining the repressed state of these genes. The NuA4 complex, the COMPASS complex, and the ISW1 complex appear to promote the expression of germ cell fate by somatic cells in the absence of the LIN-35/RB complex or the Mec complex. These complexes have been demonstrated to modify the chromatin structure that is associated with transcriptional activation. For example, the NuA4 complex contains histone acetylation activity (Cai et al., 2003; Doyon et al., 2004). Therefore, LSY-2 is involved in maintaining the repressive state of germ cell-specific genes in somatic cells. Three lines of evidence support the hypothesis that LSY-2 is likely to act in the same pathway as the Mec complex. First, loss of function of *lsy-2* results in formation of small, densely clustered P granules, and transgene silencing in somatic cells, phenotypes also observed in mutants of SUMO-recruited Mec components (Wu et al., 2012). Second, the expression profile of misexpressed P-granule components and RNAi factors in *lsy-2* mutants resembles that caused by Mec complex mutants (Wu et al., 2012). Finally, as in *lsy-2* mutants, mutations in SUMO-recruited Mec components cause the ASE asymmetry specification defect.

*lin-35* has been shown to function redundantly with other factors in specifying many developmental processes. For example, *lin-35* and *ubc-18*, which encodes an E2 ubiquitin-conjugation enzyme, function redundantly in controlling pharyngeal morphology (Fay et al., 2003; Fay et al., 2004). *lin-35* cooperates with the SWI/SNF complex (Cui et al., 2004), including *psa-1/swi3* and *xnp-1/atr-x*, and *gon-14* (encoding a protein with similarity to LIN-15B) to control larval development (Cardoso et al., 2005; Chesney et al., 2006). Here we showed that *lin-35* also functions redundantly with *lsy-2* in promoting larval development and in maintaining the soma/germ distinction. *lin-35*; *lsy-2* double mutants arrest at the early larval stage. The underlying causes of the larval arrest remain to be determined. The partially overlapping function between *lsy-2* and *lin-35* could be explained by the concerted activity of distinct complex on the same target genes or by additive effects due to misregulation of distinct genes.

## MATERIALS AND METHODS

### Strains

The following strains were used in this work: *mut-7*(*pk204*), *mut-2*(*r459*), *lsy-2*(*ot64*), *lsy-2*(*ot90*), *lin-8*(*n111*), *lin-15A*(*n767*), *lin-35*(*n745*), *lin-15B*(*n747*), *rrf-3*(*pk1426*), *lin-9*(*n112*), *hpl-2*(*ok917*), *lin-56*(*n2728*), *rrf-3*(*pk1426*), *lin-38*(*n751*), *glp-4*(*bn2*), *wIs51*(*scm*::*gfp*), *bnIs1*(*Ppie-1*::*gfp*::*gfp-1*), and *ntIs1*(*Pgcy-5*::*gfp*).

### RNAi screen

The RNAi feeding library was purchased from Geneservice. The library contains bacterial clones expressing dsRNA designed to individually inactivate 16,749 genes (targeting about 87% of the predicted genes). Synchronized L1 *gfp*::*pgl-1*; *rrf-3* animals were fed on RNAi bacterial clones and the F1 progeny or arrested larvae were examined for reporter expression.

### Indirect immunoflorescence

The monoclonal antibodies OIC1D4 and K76 for PGL-1 were obtained from the Developmental Studies Hybridoma Bank at the University of Iowa. Fragments of PGL-1 (95–551) and GLH-1 (137–572) were cloned into the pET-28a vector, expressed as His-tagged fusion proteins in *E. coli* BL21 and purified for use as an immunogen in rabbits (for PGL-1) or rat (for GLH-1).

For indirect immunofluorescence, the permeabilization of embryos and larvae was performed by freeze-cracking methods and was performed as previously described (Zhang et al., 2009).

### RNAi microinjection

Single-stranded RNA was transcribed from T7- and SP6-flanked PCR templates. The ssRNAs were then annealed and injected into young adults. F1 progeny generated four hours after injection were examined. The PCR templates used for synthesizing RNA were: *lsy-2* (F49H12, nt 33428–34151); *mut-2* (K04F10, nt 25497–26065); *mut-7* (ZK1098, nt 21677–22262); *hpl-2* (K01G5, nt 13966–14645); *lin-13* (C03B8, nt 6961–7615); *lin-65* (Y71G12B, nt 118502–119081); *lin-61* (R06C7, nt 17116–17671); *mes-4* (Y2H9A, nt 7510–8541); *isw-1* (F37A4, nt 32145–33111); *mep-1* (M04B2, nt 19879–20368); *let-418* (F26F12, nt 2286–2865); *lin-35* (C32F10, nt 7415–8214); *lin-15B* (ZK678, nt 412–873).

### RNAi feeding assay for enhanced RNAi

Gravid N2 and *lsy-2*(*ot90*) animals were grown with RNAi bacterial clones of *his-44*, *cel-1*, and vector L4440. After three hours of egg-laying, adults were taken off the plates and embryos were allowed to hatch and grow at 20°C. Animals arrested at the L3 stage was scored when worms of the same genotype fed on vector RNAi grew into adults.

### Real-time PCR assay

Synchronized L1 *glp-4* mutant animals were fed on RNAi bacterial clones at 16°C until they reached adulthood. These adults were then transferred to a new plate with the same RNAi bacterial clone and allowed to lay eggs for three hours. Adults were then taken off the plate and embryos were allowed to hatch and grow at 25°C, L4 larvae were then collected for real-time PCR.

### Measure of total SCM::GFP fluorescence intensity

Worms expressing the SCM::GFP reporter were photographed. The SCM::GFP fluorescence intensity of each seam cell in the photograph was measured by Zen 2011 and then added together to get the total SCM::GFP fluorescence intensity of the worm. Ten worms of each genotype were measured to calculate the average total SCM::GFP fluorescence intensity.

## References

[CR1] Broday L, Kolotuev I, Didier C, Bhoumik A, Gupta BP, Sternberg PW, Podbilewicz B, Ronai Z (2004). The small ubiquitin-like modifier (SUMO) is required for gonadal and uterine-vulval morphogenesis in *Caenorhabditis elegans*. Genes Dev.

[CR2] Cai Y, Jin J, Tomomori-Sato C, Sato S, Sorokina I, Parmely TJ, Conaway RC, Conaway JW (2003). Identification of new subunits of the multiprotein mammalian TRRAP/TIP60-containing histone acetyltransferase complex. J Biol Chem.

[CR3] Cardoso C, Couillault C, Mignon-Ravix C, Millet A, Ewbank JJ, Fontes M, Pujol N (2005). XNP-1/ATR-X acts with RB, HP1 and the NuRD complex during larval development in *C. elegans*. Dev Biol.

[CR4] Chesney MA, Kidd AR, Kimble J (2006). gon-14 functions with class B and class C synthetic multivulva genes to control larval growth in *Caenorhabditis elegans*. Genetics.

[CR5] Cui M, Fay DS, Han M (2004). lin-35/Rb cooperates with the SWI/SNF complex to control *Caenorhabditis elegans* larval development. Genetics.

[CR6] Cui M, Kim EB, Han M (2006). Diverse chromatin remodeling genes antagonize the Rb-involved SynMuv pathways in *C. elegans*. PLoS Genet.

[CR7] Doyon Y, Selleck W, Lane WS, Tan S, Cote J (2004). Structural and functional conservation of the NuA4 histone acetyltransferase complex from yeast to humans. Mol Cell Biol.

[CR8] Draper BW, Mello CC, Bowerman B, Hardin J, Priess JR (1996). MEX-3 is a KH domain protein that regulates blastomere identity in early *C. elegans* embryos. Cell.

[CR9] Fay DS, Yochem J (2007). The SynMuv genes of *Caenorhabditis elegans* in vulval development and beyond. Dev. Biol.

[CR10] Fay DS, Large E, Han M, Darland M (2003). lin-35/Rb and ubc-18, an E2 ubiquitin-conjugating enzyme, function redundantly to control pharyngeal morphogenesis in *C. elegans*. Development.

[CR11] Fay DS, Qiu X, Large E, Smith CP, Mango S, Johanson BL (2004). The coordinate regulation of pharyngeal development in *C. elegans* by *lin-35/Rb*, *pha-1*, and *ubc-18*. Dev Biol.

[CR12] Gruidl ME, Smith PA, Kuznicki KA, McCrone JS, Kirchner J, Roussell DL, Strome S, Benneth KL (1996). Multiple potential germ-line helicases are components of the germ-line-specific P granules of *Caenorhabditis elegans*. PNAS.

[CR13] Guedes S, Priess JR (1997). The *C. elegans* MEX-1 protein is present in germline blastomeres and is a P granule component. Development.

[CR14] Hird SN, Paulsen JE, Strome S (1996). Segregation of germ granules in living Caenorhabditis elegans embryos: cell type-specific mechanisms for cytoplasmic localisation. Development.

[CR15] Johnston RJ, Hobert O (2005). A novel *C. elegans* zinc finger transcription factor, *lsy-2*, required for the cell type-specific expression of the *lsy-6* microRNA. Development.

[CR16] Kaczynski J, Cook T, Urrutia R (2003). Sp1- and Kruppel-like transcription factors. Genome Biol.

[CR17] Kawasaki I, Shim YH, Kirchner J, Kaminker J, Wood WB, Strome S (1998). PGL-1, a predicted RNA-binding component of germ granules, is essential for fertility in *C. elegans*. Cell.

[CR18] Kunert N, Wagner E, Murawska M, Klinker H, Kremmer E, Brehm A (2009). dMec: a novel Mi-2 chromatin remodelling complex involved in transcriptional repression. Embo J.

[CR19] Kuznicki KA, Smith PA, Leung-Chiu WM, Estevez AO, Scott HC, Benneth KL (2000). Combinatorial RNA interference indicates GLH-4 can compensate for GLH-1; these two P granule components are critical for fertility in C. elegans. Development.

[CR20] Leight ER, Glossip D, Kornfeld K (2005). Sumoylation of LIN-1 promotes transcriptional repression and inhibition of vulval cell fates. Development.

[CR21] Mello CC, Schubert C, Draper B, Zhang W, Lobel R, Priess JR (1996). The PIE-1 protein and germline specification in *C. elegans* embryos. Nature.

[CR22] Ortiz CO, Faumont S, Takayama J, Ahmed HK, Goldsmith AD, Pocock R, McCormick KE, Kunimoto H, Iino Y, Lockery S, Hobert O (2009). Lateralized gustatory behavior of *C. elegans* is controlled by specific receptor-type guanylyl cyclases. Curr Biol.

[CR23] Poole RJ, Bashllari E, Cochella L, Flowers EB, Hobert O (2011). A Genome-Wide RNAi Screen for Factors Involved in Neuronal Specification in *Caenorhabditis elegans*. PLoS Genet.

[CR24] Poulin G, Dong Y, Fraser AG, Hopper NA, Ahringer J (2005). Chromatin regulation and sumoylation in the inhibition of Ras-induced vulval development in *Caenorhabditis elegans*. EMBO J.

[CR25] Robert VJ, Sijen T, van Wolfswinkel J, Plasterk RH (2005). Chromatin and RNAi factors protect the *C. elegans* germline against repetitive sequences. Genes Dev.

[CR26] Sarin S, O’Meara MM, Flowers EB, Antonio C, Poole RJ, Didiano D, Johnston RJ, Chang S, Narula S, Hobert O (2007). Genetic screens for *Caenorhabditis elegans* mutants defective in left/right asymmetric neuronal fate specification. Genetics.

[CR27] Sijen T, Plasterk RH (2003). Transposon silencing in the *Caenorhabditis elegans* germ line by natural RNAi. Nature.

[CR28] Stielow B, Sapetschnig A, Kruger I, Kunert N, Brehm A, Boutros M, Suske G (2008). Identification of SUMO-dependent chromatin-associated transcriptional repression components by a genome-wide RNAi screen. Mol Cell.

[CR29] Strome S (2005). Specification of the germ line. WormBook.

[CR30] Tabara H, Hill RJ, Mello CC, Priess JR, Kohara Y (1999). pos-1 encodes a cytoplasmic zinc-finger protein essential for germline specification in *C. elegans*. Development.

[CR31] Unhavaithaya Y, Shin TH, Miliaras N, Lee J, Oyama T, Mello CC (2002). MEP-1 and a homolog of the NURD complex component Mi-2 act together to maintain germline-soma distinctions in *C. elegans*. Cell.

[CR32] Wang D, Kennedy S, Conte D, Kim JK, Gabel HW, Kamath RS, Mello CC, Ruvkun G (2005). Somatic misexpression of germline P granules and enhanced RNA interference in retinoblastoma pathway mutants. Nature.

[CR33] Wu X, Shi Z, Cui M, Han M, Ruvkun G (2012). Repression of germline RNAi pathways in somatic cells by retinoblastoma pathway chromatin complexes. PLoS Genet.

[CR34] Zhang YX, Yan LB, Zhou Z, Yang PG, Tian E, Zhang K, Zhao Y, Li ZP, Song B, Han JH (2009). SEPA-1 mediates the specific recognition and degradation of P granule components by autophagy in *C. elegans*. Cell.

